# Detecting Lifestyle Risk Factors for Chronic Kidney Disease With Comorbidities: Association Rule Mining Analysis of Web-Based Survey Data

**DOI:** 10.2196/14204

**Published:** 2019-12-10

**Authors:** Suyuan Peng, Feichen Shen, Andrew Wen, Liwei Wang, Yadan Fan, Xusheng Liu, Hongfang Liu

**Affiliations:** 1 Center for Data Science in Health and Medicine Peking University Beijing China; 2 Department of Health Sciences Research Mayo Clinic Rochester, MN United States; 3 Institute for Health Informatics University of Minnesota Minneapolis, MN United States; 4 The Second Medical College Guangzhou University of Chinese Medicine Guangzhou China

**Keywords:** chronic kidney disease, association rule mining, Behavioral Risk Factor Surveillance System, noncommunicable diseases

## Abstract

**Background:**

The rise in the number of patients with chronic kidney disease (CKD) and consequent end-stage renal disease necessitating renal replacement therapy has placed a significant strain on health care. The rate of progression of CKD is influenced by both modifiable and unmodifiable risk factors. Identification of modifiable risk factors, such as lifestyle choices, is vital in informing strategies toward renoprotection. Modification of unhealthy lifestyle choices lessens the risk of CKD progression and associated comorbidities, although the lifestyle risk factors and modification strategies may vary with different comorbidities (eg, diabetes, hypertension). However, there are limited studies on suitable lifestyle interventions for CKD patients with comorbidities.

**Objective:**

The objectives of our study are to (1) identify the lifestyle risk factors for CKD with common comorbid chronic conditions using a US nationwide survey in combination with literature mining, and (2) demonstrate the potential effectiveness of association rule mining (ARM) analysis for the aforementioned task, which can be generalized for similar tasks associated with noncommunicable diseases (NCDs).

**Methods:**

We applied ARM to identify lifestyle risk factors for CKD progression with comorbidities (cardiovascular disease, chronic pulmonary disease, rheumatoid arthritis, diabetes, and cancer) using questionnaire data for 450,000 participants collected from the Behavioral Risk Factor Surveillance System (BRFSS) 2017. The BRFSS is a Web-based resource, which includes demographic information, chronic health conditions, fruit and vegetable consumption, and sugar- or salt-related behavior. To enrich the BRFSS questionnaire, the Semantic MEDLINE Database was also mined to identify lifestyle risk factors.

**Results:**

The results suggest that lifestyle modification for CKD varies among different comorbidities. For example, the lifestyle modification of CKD with cardiovascular disease needs to focus on increasing aerobic capacity by improving muscle strength or functional ability. For CKD patients with chronic pulmonary disease or rheumatoid arthritis, lifestyle modification should be high dietary fiber intake and participation in moderate-intensity exercise. Meanwhile, the management of CKD patients with diabetes focuses on exercise and weight loss predominantly.

**Conclusions:**

We have demonstrated the use of ARM to identify lifestyle risk factors for CKD with common comorbid chronic conditions using data from BRFSS 2017. Our methods can be generalized to advance chronic disease management with more focused and optimized lifestyle modification of NCDs.

## Introduction

Chronic kidney disease (CKD) is a progressive disease associated with high rates of mortality, morbidity, and disability [1,2]. Renal replacement therapies have been performed on approximately 8 million adults in the United States, with significant economic burdens [3]. The rate of progression of CKD from one major stage to another varies based on both unmodifiable (eg, age, race/ethnicity, family history) and modifiable (eg, hypertension, dyslipidemia, cigarette smoking, overweight/obesity, physical inactivity, dietary patterns) risk factors. Modifiable lifestyle risk factors account for 24% of the excess risk of CKD [4]. Observational and nonrandomized prospective studies have suggested that patients who modify their unhealthy lifestyles have fewer hospitalizations, are more likely to adhere to established CKD treatment goals (anemia or mineral and bone disease), and may have improved rates of survival [5-7]. Therefore, recognition of those lifestyle risk factors is vital in informing strategies to achieve renoprotection.

Lifestyle modification for CKD patients involves long-term habit changes, requires considerable effort from patients, and may take years to be effective. Evidence does exist that supports the value of lifestyle intervention for treating hypertension or diabetes and preventing cardiovascular events, but studies on suitable lifestyle interventions for patients with CKD are sparse. In addition, lifestyle risk factors for CKD with different comorbidities may vary. For example, lifestyle interventions for CKD with mineral and bone disorder include adequate calcium and vitamin D consumption, exercise, and fall prevention. The lifestyle risk factors for CKD with diabetes include unhealthy diet, sedentary lifestyle, and obesity. The lifestyle risk factors and modification strategies for CKD suggested by different guidelines may also vary [8-10], which poses a major challenge for clinical practice and research.

With the advance of digital health care strategies, a large amount of data can be leveraged for identifying lifestyle risk factors. Popular approaches for identifying lifestyle risk factors include epidemiological or statistical approaches with an implicit assumption that risk factors are linearly associated with a disease. However, it oversimplifies complex relationships between risk factors and diseases.

In this paper, we explore the use of a popular data mining technique, association rule mining (ARM), to determine more nuanced relationships between lifestyle risk factors and CKD with comorbidities. ARM is commonly used for performing unsupervised exploratory data analysis over a wide range of research and commercial domains, including biology and bioinformatics (eg, biological sequence analysis, analysis of gene expression data) [11-13]. Rules produced by ARM are able to summarize the impact of several factors in combination in a nonhierarchical fashion.

## Methods

### Materials

#### Behavioral Risk Factor Surveillance System

We conducted an ARM analysis using the 2017 Behavioral Risk Factor Surveillance System (BRFSS), which was published in July 2018 [14]. The BRFSS is an annual health-related telephone survey conducted by the Centers for Disease Control and Prevention that is designed to measure the health-related risk behaviors, chronic health conditions, and use of preventive services of adult residents (≥18 years) of the United States (including all 50 states, the District of Columbia, Guam, and Puerto Rico). More than 400,000 adults are interviewed each year, making it the largest telephone-based survey in the world and enabling it to be a powerful tool for health promotion activities. The BRFSS system consists of 29 modules and 358 variables that collect information about health status, healthy days or health-related quality of life, health care access, exercise, inadequate sleep, chronic health conditions, oral health, tobacco and e-cigarette use, alcohol consumption, immunization status, falls, seat belt use, drinking and driving, breast and cervical cancer screening, prostate cancer screening, colorectal cancer screening, and HIV/AIDS [15]. The validity of BRFSS variables for indexing chronic disease conditions has been previously demonstrated [15,16]. The BRFSS 2017 contains a total of 450,016 responses and 17,547 CKD cases.

#### Semantic MEDLINE Database

The Semantic MEDLINE Database (SemMedDB) [17] is a repository of semantic predications (subject-predicate-object triples) extracted from the titles and abstracts of all PubMed citations, which is widely used to conduct literature-based knowledge discovery in the biomedical domain [18-21]. The predications are extracted by SemRep [22], which is a semantic interpreter developed by the National Library of Medicine. Specifically, the semantic predications consist of UMLS (Unified Medical Language System) metathesaurus concepts as arguments (eg, subject and object) and a semantic relationship (eg, “treat”) from an extended version of the UMLS Semantic Network as a predicate. There are currently more than 83 million semantic predications in this database in the June 30, 2017, version of this database. Although SemMedDB provides structured predications, further inference work is needed to filter out noisy data and discover new knowledge. In this study, we treated the SemMedDB as a knowledge resource and extracted a subgraph that contains all triples related to CKD for enriching the survey data.

#### Charlson Comorbidity Index

We evaluated the noncommunicable diseases (NCDs) of each participant by using the classification of Charlson Comorbidity Index (CCI) [23], consisting of 17 comorbidities, developed and validated as a measure of 1-year mortality risk and burden of disease. In addition to CKD, we investigated five NCDs: cardiovascular disease, chronic pulmonary disease, rheumatoid arthritis, diabetes, and non-skin cancer.

Institutional review board approval was not necessary for this study due to the nature of the study (secondary analysis of an anonymized dataset).

### Methodology Overview

**Figure 1:**
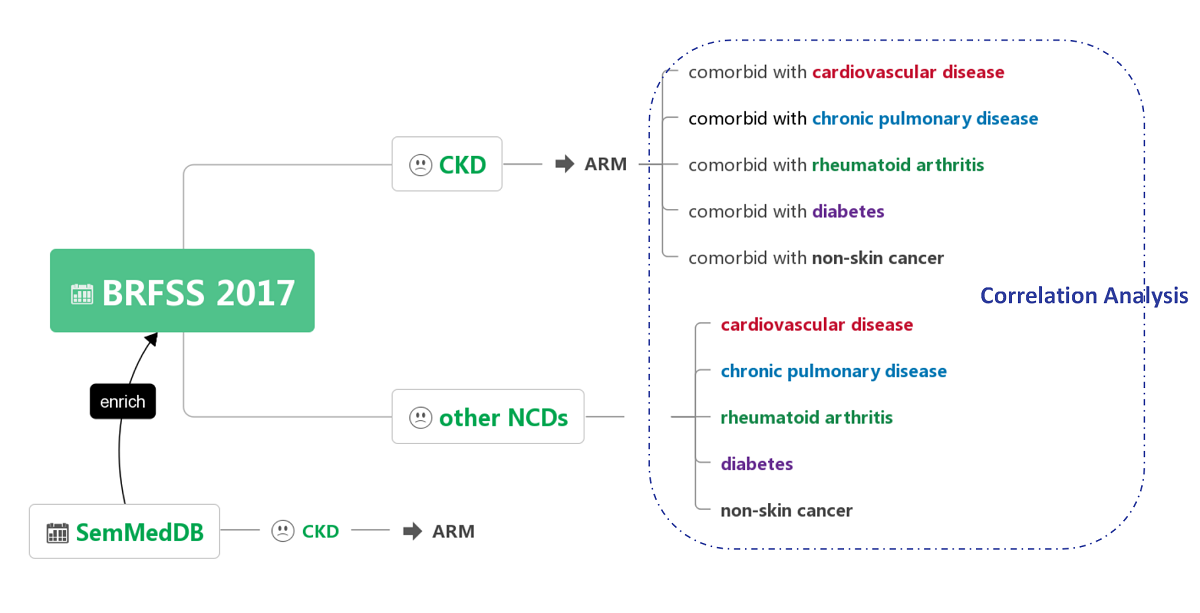
Workflow of this study. ARM: association rule mining; BRFSS: Behavioral Risk Factor Surveillance System; CKD: chronic kidney disease; NCD: noncommunicable disease; SemMedDB: Semantic MEDLINE Database.

We applied ARM for the CKD population using the 2017 BRFSS data to generate rules for detecting lifestyle risk factors for CKD progression, including demographic information, lifestyle behaviors, clinical symptoms, and chronic disease conditions. Correlation analysis was performed to assess differences in lifestyle risk factors in the status of comorbidity-related CKD. To enrich the BRFSS data, SemMedDB was mined to identify lifestyle risk factors for CKD presented in publications. The workflow is shown in Figure 1. The arules package (version v1.6-4) for R (version 3.5.2) was used for ARM analysis.

### BRFSS Input Data Preparation

We first selected 58 variables (involving 18 modules) related to behaviors from the BRFSS 2017 data by utilizing domain expert knowledge (from two nephrologists: S Peng and X Liu), with a focus on the presence of a condition or behavior rather than the questions about obvious feelings (as shown in Multimedia Appendix 1). If the given condition of interest was present in the patient, it was marked as 1, otherwise 0 or NA. For example, completion of the flu vaccine series was defined by a participant answering “yes” to the question: “During the past 12 months, have you had either a flu shot or a flu vaccine that was sprayed in your nose?” (possible answers were “yes,” “no,” “don’t know/not sure,” and “refused”). Only those who answered “yes” were annotated as 1 and included in the analysis. Records with responses of “no,” “unknown,” or “refused” were annotated as 0; those with missing data were completely excluded from the analysis to minimize underestimation. For each patient, we extracted all variables that were marked as 1 and prepared the input.

### Association Rule Mining of the Chronic Kidney Disease Cohort in BRFSS

**Figure 2:**
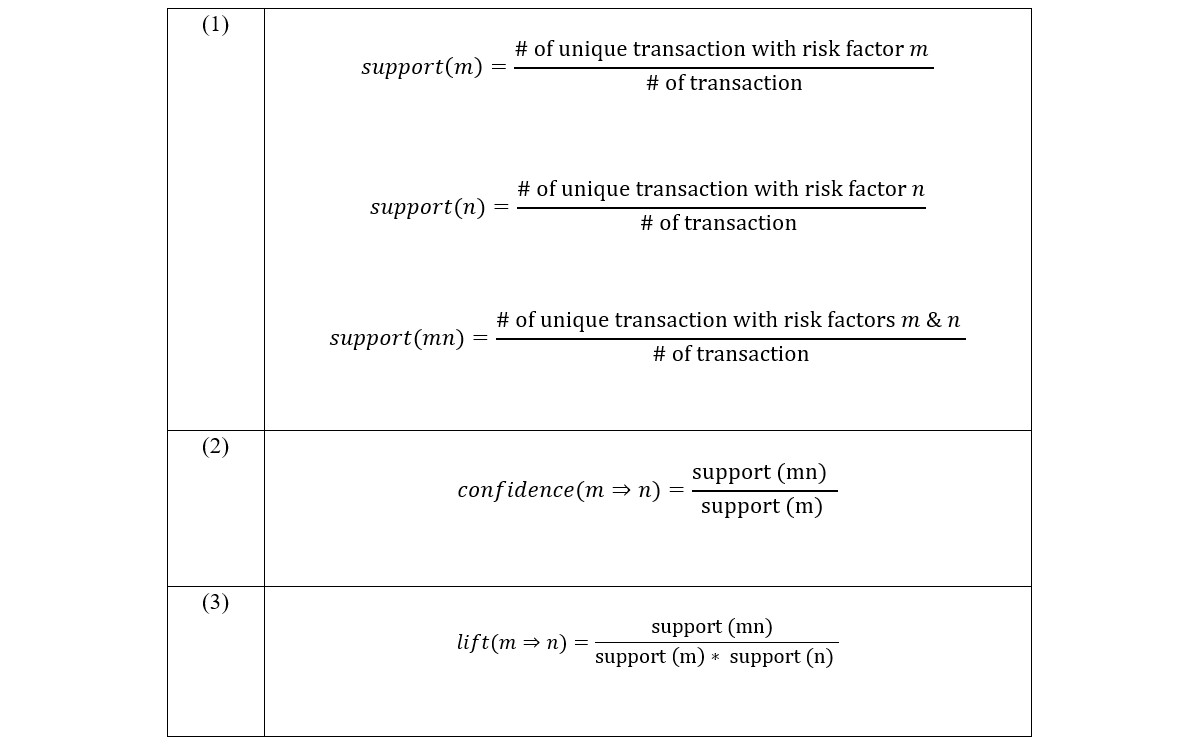
Equations.

We then applied the Apriori algorithm [24] on the input data for 58 variables among 17,547 CKD patients. Apriori is a popular algorithm for mining association rules that is divided into two steps: (1) finding frequent itemsets and (2) constructing rules from frequent itemsets. An association rule is an implication between disjoint itemsets: m ⇒ n. The left-hand side of the rule is the antecedent and the right-hand side the consequent. An itemset containing *k* items is called a *k-itemset*. If *T* is a transaction, *m* is an itemset, and m ⊆ T, then T contains m. The support of the rule m ⇒ n is the fraction of transactions that contain both m and n (equation 1 in Figure 2). A frequent itemset is one whose support is at least some threshold, always denoted as *minSup*.

The rule m ⇒ n with confidence (equation 2 in Figure 2) means that the fraction of transactions in *T* containing *m* that also contain *n* is confidence. It measures how often items in *m* appear in transactions that contain *n*. Confidence can also be referred to as the strength of the rule. The threshold of confidence is always denoted as *minConf*.

Lift (equation 3 in Figure 2) is an index that indicates the relative magnitude of the probability of observing *m* under the condition of *n*, compared with the overall probability of observing *m*. When lif*t* = 1, the two occurrences, m ⇒ n, are independent of each other. When the lift value is greater than 1, the two occurrences are dependent on one another; the higher the value, the greater the relevance of the interaction.

We used the following heuristic for generating the final association rules to be analyzed. We first selected itemsets with support value larger than the average of support values of all itemsets and lift value greater than 1. We then kept itemsets with lift value larger than the average lift values of those selected itemsets as our final association rules. We focused our analysis of the association rules of five NCDs as determined by the CCI: cardiovascular disease, chronic pulmonary disease, rheumatoid arthritis, diabetes, and non-skin cancer.

### Correlation Analysis of Comorbidities and Risk Factors

To assess differences of lifestyle risk factors in the status of comorbidity-related CKD, correlation analysis was performed. We retrieved five subcohorts of NCDs (cardiovascular disease, chronic pulmonary disease, rheumatoid arthritis, diabetes, non-skin cancer) from the CKD cohort and evaluated the contribution and correlation of lifestyle risk factors.

### Literature Enrichment Analysis

We mined SemMedDB for lifestyle risk factors that were not present in the BRFSS system by also using ARM. We first retrieved all relevant triplets (subject, predicate, and object) related to CKD. For example, <Diet Therapy, Treats, CKD>, <CKD, Coexists_With, Diabetes>, and <CKD, Causes, Hypertensive Disease> are some example triplets retrieved. We then selected terms from the triplets that were relevant to lifestyle behavior, symptoms, and diseases based on a list of relevant semantic types (see Multimedia Appendix 2). We filtered out 47 terms with generic meaning (eg, patients, agent, woman, child, author, disease).

We then applied the Apriori algorithm on the extracted pairwise terms to mine frequent itemsets and generate rules. Based on our previous work [25], our item matrix was very sparse, with a density of 0.00026. To mine sufficiently interesting rules, we set the minimum support and minimum confidence by making sure every rule was presented at least two times and selected itemsets with lift value greater than 1. We then kept itemsets with lift value larger than the average lift values of those selected itemsets as our final association rules. Specifically, we focused our analysis of the association rules on six specific semantic types (daily or recreational activity, food, hazardous or poisonous substance, individual behavior, mental or behavioral dysfunction, finding) to detect lifestyle risk factors present in publications that the BRFSS questionnaire does not mention.

## Results

### Characteristics of Patients With Chronic Kidney Disease Cohort

Overall, a total of 17,547 participants were reported have CKD in the BFRSS 2017 data; 80.09% (14,053/17,547) were white and 60.13% (10,551/17,547) were men. The mean age was 64.42 (SD 13.81) years. The characteristics of the CKD cohort are presented in Table 1.

**Table 1:** Characteristics of participants in the BFRSS (Behavioral Risk Factor Surveillance System) 2017 with chronic kidney disease (N=17,547).

Characteristics		Participants
Age (years), mean (SD)		64.42 (13.81)
Male, n (%)		10,551 (60.13)
Completed interview, n (%)		15,348 (87.47)
Ever served on active duty in the United States Armed Forces, n (%)		2940 (16.76)
**Income categories^a^, n (%)**		
	Less than $15,000	2608 (14.86)
	$15,000 to less than $25,000	3455 (19.69)
	$25,000 to less than $35,000	1792 (10.21)
	$35,000 to less than $50,000	1955 (11.14)
	$50,000 or more	4734 (26.98)
**Education level, n (%)**		
	Did not graduate middle school	1933 (11.02)
	Did not graduate high school	5213 (29.71)
	Attended college or technical school	5151 (29.36)
	Graduated from college or technical school	5181 (29.53)
**Marital status, n (%)**		
	Married	7904 (45.04)
	Divorced	3047 (17.36)
	Widowed	3821 (21.78)
	Separated	494 (2.82)
	Never married	1801 (10.26)
	A member of an unmarried couple	374 (2.13)
**Race, n (%)**		
	White	14,053 (80.09)
	Black or African American	1763 (10.05)
	American Indian or Alaskan Native	535 (3.05)
	Asian	261 (1.49)
	Native Hawaiian or other Pacific Islander	159 (0.91)
	Other race	351 (2.00)
	No preferred race	50 (0.28)
**Comorbidity, n (%)**		
	CHD^b^ or myocardial infarction	4828 (28.12)
	Stroke	15,204 (86.65)
	COPD^c^, emphysema, or chronic bronchitis	3763 (21.45)
	Asthma	2888 (16.46)
	Rheumatoid arthritis	10,798 (61.98)
	Diabetes	6642 (37.85)
	Cancer	3974 (22.65)

^a^The rest of the people refused to answer this question.

^b^CHD: coronary heart disease.

^c^COPD: chronic obstructive pulmonary disease.

### Association Rule Mining of the Chronic Kidney Disease Cohort in BRFSS

**Figure 3:**
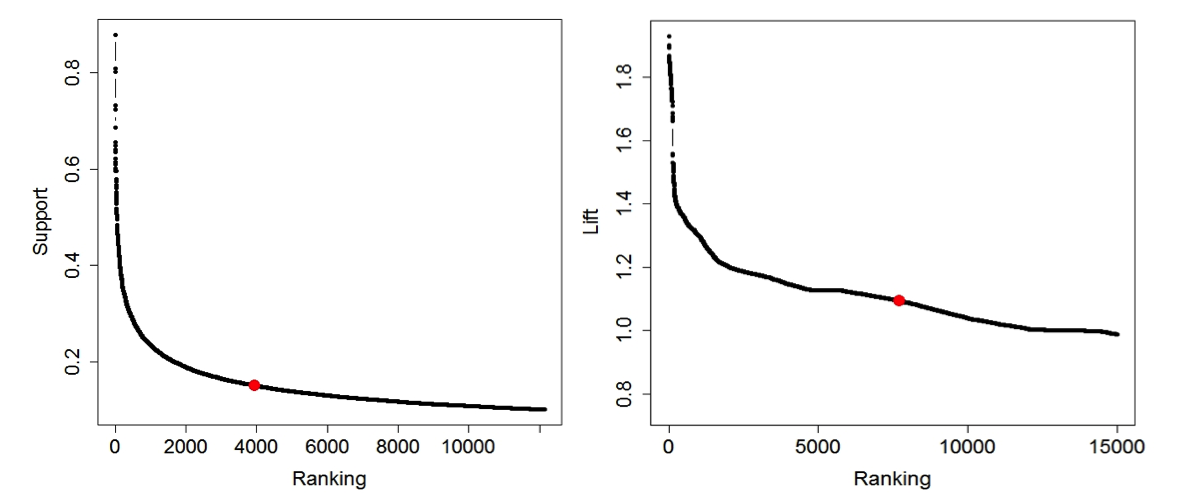
Support and lift value selection.

For heuristics, we set a lower bound of 0.1 for support and computed the average for all selected support values. As a result, we set the average support (0.150) as a threshold and selected 12,141 frequent itemsets. Among the 12,141 frequent itemsets, we then picked the average lift 1.094 as the threshold to finalize 7677 association rules. Figure 3 shows the curve between ranked associations and interestingness metrics (support and lift). The threshold was also marked on the curve.

Among the 7677 association rules, we retrieved subsets that related to five adverse conditions included in NCDs from CCI, including cardiovascular disease, chronic pulmonary disease, rheumatoid arthritis, diabetes, and non-skin cancer. For each of the input conditions, we then selected the top 10 association rules with the highest lift score regardless of whether the disease appeared on the left or right side.

From the top rules of each comorbidity, we determined that (1) CKD patients with comorbidity of cardiovascular disease have symptoms of high blood pressure, high cholesterol, asthma, function limitation, and lower aerobic and strengthening level; (2) CKD patients with a comorbidity of chronic pulmonary disease tend to have clinical manifestations of being overweight, hypertension, unhealthy diet (french fries or fried potatoes, less consumption of fruit and vegetables), and lower aerobic and strengthening level; (3) CKD patients with a comorbidity of rheumatoid arthritis are associated with hypertension, overweight, asthma, difficulty walking/doing errands alone, and less leisure-time physical activities; (4) CKD patients with a comorbidity of diabetes have a variety of clinical manifestations, including hypertension, high cholesterol, overweight, less leisure-time physical activities, and lower aerobic and strengthening level; and (5) CKD patients with non-skin cancer are associated with age (older than 65 years), asthma, less muscle strengthening, and lower aerobic level.

Examples of the top rules with the highest lift scores are shown in Table 2 (see Multimedia Appendix 3 for details on the results of the top 10 rules for subsets).

**Table 2:** Examples of the top rule for subsets.

Comorbidities	Keywords^a^	Top rule	Lift	Count
Cardiovascular disease	‘x.michd’	{diffwalk,x.casthm1,x.rfhype5,x.rfsmok3} => {x.michd}	1.43	2783
Chronic pulmonary disease	chccopd1,’ ‘x.casthm1’	{diffalon,x.casthm1,x.drdxar1,x.rfsmok3} => {diffwalk}	1.93	2643
Rheumatoid arthritis	‘x.drdxar1’	{diffalon,x.casthm1,x.drdxar1,x.rfsmok3} => {diffwalk}	1.93	2676
Diabetes	‘diabete3’	{diffwalk,x.casthm1,x.rfchol1,x.rfhype5,x.rfsmok3} => {diabete3}	1.53	2726
Cancer	‘chcocncr’	{chcocncr,x.casthm1} => {x.age65yr}	1.2	2697

^a^We used the variable code to represent each variable. The meaning of the code is shown in Multimedia Appendix 1.

### Correlation Analysis of Comorbidities and Risk Factors

**Figure 4:**
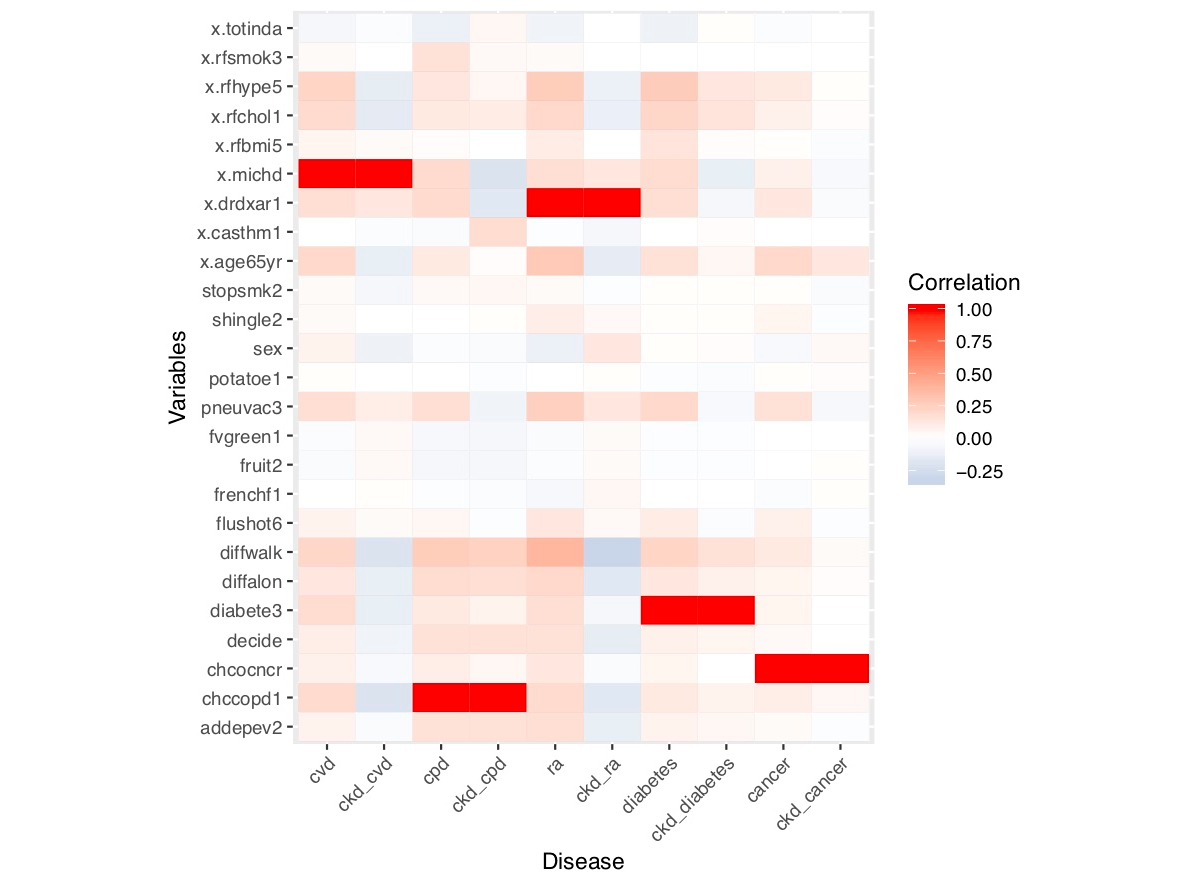
Heatmap for correlation analysis of comorbidities and lifestyle risk factors.

We conducted a correlation analysis using variables present in the top 10 rules for the five NCDs and CKD with a total of 25 variables. Figure 4 shows the heatmap of the correlation coefficient values of those variables with the five NCDs, CKD, and CKD with the comorbidities. Spearman rank correlation test was used for the analysis.

The correction analysis showed that people with NCDs including CKD have less physical activity in their leisure time and consume fewer fruits and vegetables. Hypertension, high cholesterol, age older than 65 years, male sex, difficulty walking, and difficulty concentrating had positive correlations with cardiovascular disease and rheumatoid arthritis but negative correlations with CKD comorbid conditions.

### Literature Enrichment Analysis

We set the support threshold to 0.00047 and the confidence threshold to 0.0001 to ensure every rule was presented at least two times, and then expressed the results with lift value greater than 1 by descending order to finalize important association rules. Among all 1323 association rules, 140 keywords from six specific semantic types were selected as a lifestyle word list (daily or recreational activity, food, hazardous or poisonous substance, individual behavior, mental or behavioral dysfunction, finding) to detect novel rules related to lifestyle risk factors present in publications. Multimedia Appendix 4 shows the top 20 rules.

Associations found using this method indicated that iron deficiency, depressed mood, sedentary lifestyle, and malnutrition were associated with anemia, hyperparathyroidism, obesity, and atherosclerosis, respectively, which the BRFSS questionnaire does not mention (Table 3).

**Table 3:** Lifestyle-related top rules of SemMedDB.

Top rules	Lift	Count
{Iron deficiency} => {Anemia}	20.92	3
{Depressed mood} => {Hyperparathyroidism; Secondary}	16.24	2
{Obesity} => {Sedentary}	15.18	2
{Obesity} => {Hypercholesterolemia}	15.18	2
{Malnutrition} => {Atherosclerosis}	9.33	3

## Discussion

### Comparison With Other Studies and Reviews

The association rules indicated that CKD patients comorbid with cardiovascular disease are more likely to have symptoms of high blood pressure, high cholesterol, asthma, function limitation, and lower aerobic and strengthening levels.

The proper assessment of overall progressive risk in patients with CKD requires an adequate assessment of the presence and severity of other major risk factors. CKD is an independent risk factor for the development of cardiovascular disease; CKD is considered a cardiovascular disease risk equivalent [26,27]. Damaged kidneys may release too much renin, which helps to control blood pressure but increases the risk for heart attack, congestive heart failure (CHF), and stroke. CHF is responsible for up to 50% of deaths in patients with renal failure [3,28]. The signs and symptoms of heart failure include shortness of breath (dyspnea), fatigue, and weakness, consistent with our findings. The Physicians’ Health Study and other observational studies suggest that increased physical activity, higher cardiorespiratory fitness, and lower sedentary time are associated with reduced incidence of CHF [29]. Evidence shows that exercise training results in improved physical performance and functioning in patients with CKD [30]. Hence, the highlight of lifestyle modification of CKD with cardiovascular disease is to increase aerobic capacity by improving muscle strength or functional ability.

Findings also point to similar risk factors for CKD with chronic pulmonary disease or rheumatoid arthritis. The relationship between rheumatoid arthritis and chronic pulmonary disease (especially for chronic obstructive pulmonary disease) was found recently, in which people with rheumatoid arthritis were at 47 percent greater risk of hospitalization for chronic obstructive pulmonary disease than those in the control group [31]. Our finding supported evidence in the CKD cohort, the mechanisms that link CKD comorbid with the two diseases are speculative at present, which might be inflammation, autoimmunity, or genetic predispositions shared between them.

The lifestyle risk factors of these two comorbidities include hypertension, overweight, unhealthy diet (french fries or fried potatoes, less consumption of fruit and vegetables), and physical inactivity. Because the items did not determine exactly when symptoms of CKD or other NCDs originated, there are two possible interpretations of the result. One possible interpretation is that participants began reducing their physical activity and intake of fruits and vegetables when they developed CKD or chronic conditions. Symptoms of chronic conditions, such as hypertension, bone pain, peripheral neuropathy, side effects from medicines and fluid retention, itch, or sleep disturbance, can all negatively affect daily physical activity level, especially for CKD patients. Fruits and vegetables are a rich source of carbohydrates, vitamins, potassium, magnesium, and dietary fiber, whereas legumes and dried beans are important vegetable proteins. However, the limitation of potassium, fructose [32,33], or dietary protein intake has been common practice to control uremia. Despite the known benefits of fruit and vegetable consumption, intake remains poor in both the general and CKD populations [34].

An alternative interpretation is that lower vegetable and fruit consumption contributes to the development or maintenance of CKD or other NCDs. This interpretation has greater plausibility because it is consistent with other epidemiological studies and existing biological knowledge. However, fruits and vegetables should not be omitted from the everyday diet; this practice may lead to nutrient deficiency and low fiber-related constipation, which contribute to further accumulation of uremic toxins. The national “2 fruits and 5 vegetables” campaign guides Australians toward healthy fruit and vegetable consumption, which is applicable to CKD [35]. Also, regular participation in moderate-intensity exercise may enhance certain aspects of immune function and exert anti-inflammatory effects. Therefore, the lifestyle modification of CKD with chronic pulmonary disease or rheumatoid arthritis should be high dietary fiber intake and participation in moderate-intensity exercise to decrease inflammation and oxidative stress.

CKD is associated with insulin resistance and, in advanced CKD, decreased insulin degradation. In the association rules for CKD with diabetes, the results pointed to hypertension, high cholesterol, overweight, less leisure-time physical activities, and lower aerobic and strengthening as lifestyle risk factors rather than an unhealthy diet. Hence, the lifestyle modification of CKD with diabetes is consistent with the prevention of type 2 diabetes (predominantly exercise and weight loss), which can successfully decrease the development of CKD with diabetes.

CKD is recognized as a disease that may complicate cancer and its therapy (eg, immunotherapy). Cancer can cause CKD either directly or indirectly through the adverse effects of therapies; conversely, CKD may be a risk factor for cancer [36,37]. We found that age older than 65 years and physical inactivity were associated with CKD with non-skin cancer. The BRFSS questionnaire does not incorporate the therapeutics of cancer; therefore, the lifestyle risk factors of CKD with cancer cannot be evaluated in our research.

### Enrichment of the BRFSS Questionnaire

The BRFSS does not specifically target CKD or NCDs; therefore, many clinical manifestations were not considered, including potentially relevant items such as anorexia, nausea, vomiting, fatigue, anemia, and bone disease. To enrich the questionnaire, we used the SemMedDB to find lifestyles that related to the clinical manifestations specifically with CKD from publications. The results indicated that iron deficiency, depressed mood, sedentary lifestyle, and malnutrition are associated with anemia, hyperparathyroidism, obesity, and atherosclerosis, respectively, which the BRFSS questionnaire did not mention. CKD can affect a patient’s health-related quality of life in many ways. The diagnosis alone might cause fear or anxiety. Anemia, frailty, coexisting comorbidities, and depression are also major contributory factors to quality of life in CKD. Meat and meat alternatives are the main source of protein in the CKD diet. Healthy choices include lean cuts of meat, skinless poultry, eggs, fish, seafood, and plant-based protein foods such as legumes, dried beans, nuts, and seeds. The questionnaire of the BRFSS does not contain the variables of meat or protein consumption, nor does it contain information on micronutrient deficiency.

### Effectiveness of Association Rule Mining in the Noncommunicable Disease Domain

The results of the correlation analysis found that hypertension, high cholesterol, age older than 65 years, male sex, difficulty walking, and attention deficit disorder were positively correlated with cardiovascular disease and rheumatoid arthritis, but negatively correlated with corresponding CKD comorbidities (CKD with cardiovascular disease/rheumatoid arthritis). The ARM results suggest that patients with CKD older than 65 years are more likely to have signs or symptoms of hypertension, asthma, and difficulty walking, which is inconsistent with the aforementioned findings. It was caused by the differences between the two algorithms: a correlation is the relationship that exists between two or more variables in which a change in one variable causes a change in the other variable when the two variables are said to be correlated. Association rules are of the form {X_1_, ..., X_n_} → Y, meaning that if you find all signs or symptoms of X_1_, ..., X_n_ in a disease it is possible to find another sign or symptom (Y). Epidemiological studies and existing domain knowledge are inconsistent with the result of correlation analysis but consistent with the results of ARM.

A wide range of disorders may develop as a consequence of the loss of renal function with CKD. These include disorders of fluid and electrolyte balance, as well as abnormalities related to hormonal or systemic dysfunction. Treatment strategies should be modified based on the needs of the individual patient. Variations and inconsistencies are inevitable in clinical practice; therefore, recognizing modifiable risk factors in medical interventions are important for providing effective chronic disease management. ARM has several applications in the medical domain, and it has been used for detecting risk factors for diabetes and cardiovascular disease [38,39]. This study illustrates how ARM approaches could be used in risk factor detection of CKD and provides the potential effectiveness of the method of ARM analysis for NCDs. ARM methods, such as Apriori, have also been used on electronic health record data to identify associations among clinical concepts. The strength of the ARM approach compared with a more conventional correlation analysis is that it has identified sizeable groups that can easily be defined and identified for intervention at a practice level in real time to allow more focused and immediate correction of bias in chronic disease management.

### Limitations

This research used a large representative sample, was based on items that asked about diagnosed disease, and included a number of relevant covariates; however, there are some aspects of this study that should be noted as limitations.

First, from 17,547 CKD patients, only 15,348 completed the interview. The dataset was skewed toward the white race and male gender, which may affect the generalizability of lifestyle interventions to other races and females. Other research found similar results [40] in which lower response rates (<40%) were associated with the underrepresentation of racial/ethnic minorities (eg, Hispanics), women, and younger individuals in the BRFSS survey.

Second, CKD and cancer can influence each other either directly or indirectly through the adverse effects of therapies. Since the BRFSS questionnaire was based on self-reporting, we cannot connect enough information. The lifestyle risk factors of CKD with cancer could be confirmed in further research using direct physical examination or biochemical indexes.

Third, the semantic predications consist of UMLS metathesaurus concepts as arguments, so we cannot tell whether “sedentary lifestyle” and “depressed mood” can be treated as “leisure-time physical activity calculate variables” or “ever been told you have depressive disorder.” As such, whether these differences are involved in observed associations for CKD needs to be considered in further epidemiological research. More robust observational or quasi-experimental studies would be needed to fully support the long-term impact of interventions for modifiable risk factors.

Finally, for the semantic predication triples extracted from the SemMedDB, we ignored the semantic meaning of the predicates and only kept subjects and objects as pairwise associations. However, we also found some predications with negative meanings. For example, the triples <Diet; Protein-Restricted, Neg_Treats, CKD>, <Dietary intake, Neg_Associated_With, CKD>, <End-stage renal failure, Neg_Coexists_With, CKD>, and <Ferritin level, Neg_Manifestation_Of, CKD> contain predicates with negative meaning, like not treats, have no association with, does not coexist with, or does not manifest. The reason we did not completely remove those triples is that we found inconsistency because both positive and negative relationships for the same factor may be reported. For example, according to a 2015 study conducted by Wong [41], a positive relationship between abnormal blood pressure and CKD was found in SemMedDB; however, in a 1992 study conducted by Taniguchi et al [42], a negative relationship between the same two items was detected. The SemMedDB only maintains information contained in the title and abstract; therefore, it is difficult to address inconsistencies without reading through the full text. In the future, we will count positive and negative associations for each pairwise term and assign weights for different predications for a better semantic representation.

### Conclusion

This study related both lifestyle risk factors and CKD with five other comorbid chronic conditions using the largest national US survey available and provided a suggestion for BRFSS questionnaire enrichment. Various lifestyle risk factors result in the presence of different comorbid conditions for CKD patients, and different signs and symptoms may be observed. The findings illustrate how ARM approaches could be used in risk factor detection of chronic diseases to allow more focused and optimized chronic disease management.

## Appendix

Multimedia Appendix 1 BRFSS 2017 code mapping index.

Multimedia Appendix 2 Selected semantic types.

Multimedia Appendix 3 Top 10 rules for subsets.

Multimedia Appendix 4 Lifestyle-related rules from SemMedDB.

## Abbreviations

ARM: association rule mining
BRFSS: Behavioral Risk Factor Surveillance System
CCI: Charlson Comorbidity Index
CHF: congestive heart failure
CKD: chronic kidney disease
NCDs: noncommunicable diseases
SemMedDB: Semantic MEDLINE Database
UMLS: Unified Medical Language System
