# Supplementary material for: Detecting Lifestyle Risk Factors for Chronic Kidney Disease With Comorbidities: Association Rule Mining Analysis of Web-Based Survey Data
Source: J Med Internet Res. 2019 Dec 10;21(12):e14204. doi: 10.2196/14204 (PMC6930505; doi:10.2196/14204)
Supplement: Multimedia Appendix 1 [file jmir_v21i12e14204_app1.docx]

**BRFSS 2017 Code Mapping Index**

| **Variable Name** | **Question** | **Section Name** |
| --- | --- | --- |
| cvdstrk3 | ever diagnosed with a stroke | Chronic Health Conditions |
| chcscncr | (ever told) you had skin cancer? | Chronic Health Conditions |
| chcocncr | (ever told) you had any other types of cancer? | Chronic Health Conditions |
| chccopd1 | (ever told) you have chronic obstructive pulmonary disease, emphysema or chroic bronchitis? | Chronic Health Conditions |
| addepev2 | ever told you had a depressive disorder | Chronic Health Conditions |
| chckidny | (ever told) you have kidney disease? | Chronic Health Conditions |
| diabete3 | (ever told) you have diabetes | Chronic Health Conditions |
| sex | respondents sex | Demographics |
| pregnant | pregnancy status | Demographics |
| deaf | are you deaf or do you have serious difficulty hearing? | Demographics |
| blind | blind or difficulty seeing | Demographics |
| decide | difficulty concentrating or remembering | Demographics |
| diffwalk | difficulty walking or climbing stairs | Demographics |
| diffdres | difficulty dressing or bathing | Demographics |
| diffalon | difficulty doing errands alone | Demographics |
| stopsmk2 | stopped smoking in past 12 months | Tobacco Use |
| fruit2 | how many times did you eat fruit? | Fruits & Vegetables |
| fruitju2 | how many times did you drink 100 percent pure fruit juices? | Fruits & Vegetables |
| fvgreen1 | how many times did you eat dark green vegetables? | Fruits & Vegetables |
| frenchf1 | how often do you eat french fries or fried potatoes? | Fruits & Vegetables |
| potatoe1 | how often do you eat potatoes | Fruits & Vegetables |
| flushot6 | adult flu shot/spray past 12 mos | Immunization |
| pneuvac3 | pneumonia shot ever | Immunization |
| shingle2 | have you ever had the shingles or zoster vaccine? | Immunization |
| insulin | now taking insulin | Diabetes |
| copdcogh | did you have a cough? | Respiratory Health (COPD Symptoms) |
| copdflem | did you cough up phlegm? | Respiratory Health (COPD Symptoms) |
| copdbrth | did you have shortness of breath? | Respiratory Health (COPD Symptoms) |
| cvdasprn | take aspirin daily or every other day | Cardiovascular Health |
| bpsalt | reduce salt intake to lower BP? | Actions to Control High Blood Pressure |
| bpalchol | reduce alcohol use for BP? | Actions to Control High Blood Pressure |
| bpexer | exercising to lower BP? | Actions to Control High Blood Pressure |
| adsleep | days had trouble with sleep | Sleep Disorder |
| slepsno2 | have you ever been told that you snore loudly? | Sleep Disorder |
| slepbrth | has anyone ever observed that you stop breathing during your sleep? | Sleep Disorder |
| csrvctl1 | is pain under control? | Cancer Survivorship |
| ssbsugr2 | how often did you drink regular soda or pop that contains sugar? | Sugar Sweetened Beverages |
| ssbfrut3 | how often did you drink sugar-sweetened drinks? | Sugar Sweetened Beverages |
| wtchsalt | watching sodium or salt intake | Sodium or Salt-Related Behavior |
| marijana | during the past 30 days, on how many days did you use marijuana or hashish? | Marijuana |
| hpvadvc2 | have you ever had the HPV vaccination? | Adult Human Papilloma Virus (HPV) |
| tetanus | received tetanus shot since 2005? | Tetanus, Diphtheria, and Acellular Pertussis (Tdap) (Adults) |
| cimemlos | have you experienced confusion or memory loss that is happening more often or is getting worse? | Cognitive Decline |
| sxorient | sexual orientation or gender identity | Sexual Orientation and Gender Identity |
| trnsgndr | do you consider yourself to be transgender? | Sexual Orientation and Gender Identity |
| x.rfhype5 | high blood pressure calculated variable | Calculated Variables |
| x.rfchol1 | high cholesterol calculated variable | Calculated Variables |
| x.michd | ever had CHD or MI | Calculated Variables |
| x.casthm1 | current asthma calculated variable | Calculated Variables |
| x.drdxar1 | respondents diagnosed with arthritis | Calculated Variables |
| x.prace1 | computed preferred race | Calculated Variables |
| x.age65yr | reported age in two age groups calculated variable | Calculated Variables |
| x.rfbmi5 | overweight or obese calculated variable | Calculated Variables |
| x.rfsmok3 | current smoking calculated variable | Calculated Variables |
| x.rfdrhv5 | heavy alcohol consumption calculated variable | Calculated Variables |
| x.totinda | leisure time physical activity calculated variable | Calculated Variables |
| x.pastrng | muscle strengthening recommendation | Calculated Variables |
| x.pastae1 | aerobic and strengthening (2-level) | Calculated Variables |
| x.aidtst3 | ever been tested for HIV calculated variable | Calculated Variables |
