# Supplementary material for: Detecting Lifestyle Risk Factors for Chronic Kidney Disease With Comorbidities: Association Rule Mining Analysis of Web-Based Survey Data
Source: J Med Internet Res. 2019 Dec 10;21(12):e14204. doi: 10.2196/14204 (PMC6930505; doi:10.2196/14204)
Supplement: Multimedia Appendix 2 [file jmir_v21i12e14204_app2.docx]

**Selected Semantic Types**

| **Type Code** | **Semantic Types** | **Example of UMLS Concepts** |
| --- | --- | --- |
| aggp | Age Group | Elderly; Adolescent; Octogenarians; etc. |
| bird | Bird | Dipper; Meleagris gallopavo; |
| bpoc | Body Part, Organ, or Organ Component | Arteries; Bladder; Colon; etc. |
| carb | Carbohydrate | Carbohydrates; Low-Molecular-Weight; etc. |
| clna | Clinical Attribute | Body mass index; Diastolic blood pressure; etc. |
| dora | Daily or Recreational Activity | Cigarette Smoking; Exercise; Therapeutic diets; etc. |
| dsyn | Disease or Syndrome | Abdominal obesity; Abdominal obesity; etc. |
| fish | Fish | Oncorhynchus mykiss; Mola; .etc |
| fndg | Finding | Body Weight Changes; Encounter due to tobacco use; .etc |
| food | Food | Fruit; Poultry Meat; Nut; .etc |
| hops | Hazardous or Poisonous Substance | Illicit Drugs; Opioids; Warfarin; .etc |
| humn | Human | Households; Black Race; Polish population; .etc |
| inbe | Individual Behavior | Alcohol consumption; intravenous drug use; .etc |
| lipd | Lipid | Dietary Fats; Phospholipids; .etc |
| mobd | Mental or Behavioral Dysfunction | Major Depressive Disorder; Major Depressive Disorder; .etc |
| moft | Molecular Function | antioxidant activity; Receptor Binding; .etc |
| npop | Natural Phenomenon or Process | Electromagnetic Energy; Physical Dialysis; .etc |
| orga | Organism Attribute | Heart rate; Myocardial perfusion; .etc |
| orgf | Organism Function | fibrogenesis; Wound Healing; .etc |
| ortf | Organ or Tissue Function | Cardiac function; Immunologic function; .etc |
| sosy | Sign or Symptom | Drowsiness; Dyspnea; Edema of lower extremity; .etc |
| neop | Neoplastic Process | Polycythemia Vera; Malignant neoplasm of kidney; .etc |
| anab | Anatomical Abnormality | Cataract; pathologic fistula; pathologic fistula; .etc |
| cgab | Congenital Abnormality | Congenital Abnormality; Skin Abnormalities; .etc |
| inpo | Injury or Poisoning | Adverse drug effect; Brain Injury; .etc |
