# Supplementary material for: Detecting Lifestyle Risk Factors for Chronic Kidney Disease With Comorbidities: Association Rule Mining Analysis of Web-Based Survey Data
Source: J Med Internet Res. 2019 Dec 10;21(12):e14204. doi: 10.2196/14204 (PMC6930505; doi:10.2196/14204)
Supplement: Multimedia Appendix 3 [file jmir_v21i12e14204_app3.docx]

**Top 10 Rules for Subsets**

| **Comorbidities** | **Keywords** | **Top 10 Rules** | **Sup** | **Con** | **Lift** | **Count** |
| --- | --- | --- | --- | --- | --- | --- |
| Cardiovascular Disease | 'x.michd'^a^ | {diffwalk,x.casthm1,x.rfhype5,x.rfsmok3} => {x.michd} | 0.15 | 0.39 | 1.43 | 2564 |
|  |  | {x.casthm1,x.michd} => {diabete3} | 0.15 | 0.54 | 1.41 | 2565 |
|  |  | {x.michd,x.rfhype5,x.rfsmok3} => {diffwalk} | 0.15 | 0.65 | 1.39 | 2583 |
|  |  | {x.casthm1,x.michd,x.rfhype5,x.rfsmok3} => {diffwalk} | 0.15 | 0.65 | 1.39 | 2564 |
|  |  | {x.casthm1,x.michd,x.rfsmok3} => {diffwalk} | 0.17 | 0.64 | 1.37 | 2973 |
|  |  | {x.casthm1,x.michd} => {diffwalk} | 0.17 | 0.63 | 1.33 | 3004 |
|  |  | {x.casthm1,x.rfchol1,x.rfhype5,x.rfsmok3} => {x.michd} | 0.16 | 0.36 | 1.32 | 2889 |
|  |  | {x.michd,x.rfhype5,x.rfsmok3} => {x.rfchol1} | 0.17 | 0.74 | 1.3 | 2915 |
|  |  | {x.casthm1,x.michd,x.rfhype5,x.rfsmok3} => {x.rfchol1} | 0.16 | 0.74 | 1.3 | 2889 |
|  |  | {pneuvac3,x.casthm1,x.michd,x.rfsmok3} => {x.age65yr} | 0.15 | 0.74 | 1.29 | 2579 |
| Chronic pulmonary disease | 'chccopd1',  'x.casthm1' | {diffalon,x.casthm1,x.drdxar1,x.rfsmok3} => {diffwalk} | 0.15 | 0.91 | 1.93 | 2621 |
|  |  | {diffalon,x.casthm1,x.rfhype5,x.rfsmok3} => {diffwalk} | 0.15 | 0.89 | 1.89 | 2640 |
|  |  | {fvgreen1,potatoe1,x.casthm1,x.rfbmi5,x.rfsmok3} => {frenchf1} | 0.16 | 0.64 | 1.87 | 2728 |
|  |  | {flushot6,pneuvac3,x.age65yr,x.casthm1,x.rfsmok3} => {shingle2} | 0.17 | 0.54 | 1.83 | 2970 |
|  |  | {frenchf1,fruit2,fvgreen1,x.casthm1,x.rfsmok3} => {potatoe1} | 0.20 | 0.78 | 1.83 | 3439 |
|  |  | {fruit2,fvgreen1,potatoe1,x.casthm1,x.rfsmok3} => {frenchf1} | 0.20 | 0.62 | 1.82 | 3439 |
|  |  | {fvgreen1,potatoe1,x.casthm1,x.rfhype5,x.rfsmok3} => {frenchf1} | 0.16 | 0.62 | 1.82 | 2748 |
|  |  | {fvgreen1,pneuvac3,x.age65yr,x.casthm1,x.rfsmok3} => {shingle2} | 0.15 | 0.53 | 1.81 | 2715 |
|  |  | {pneuvac3,x.age65yr,x.casthm1,x.rfchol1,x.rfsmok3} => {shingle2} | 0.15 | 0.53 | 1.81 | 2591 |
|  |  | {fruit2,potatoe1,x.casthm1,x.rfbmi5,x.rfsmok3} => {frenchf1} | 0.16 | 0.62 | 1.81 | 2830 |
| Rheumatoid Arthritis | 'x.drdxar1' | {diffalon,x.casthm1,x.drdxar1,x.rfsmok3} => {diffwalk} | 0.15 | 0.91 | 1.93 | 2621 |
|  |  | {diffwalk,x.drdxar1,x.rfsmok3} => {diffalon} | 0.15 | 0.42 | 1.9 | 2653 |
|  |  | {diffwalk,x.drdxar1} => {diffalon} | 0.15 | 0.42 | 1.9 | 2676 |
|  |  | {diffwalk,x.casthm1,x.drdxar1,x.rfsmok3} => {diffalon} | 0.15 | 0.42 | 1.9 | 2621 |
|  |  | {diffwalk,x.casthm1,x.drdxar1} => {diffalon} | 0.15 | 0.42 | 1.9 | 2643 |
|  |  | {pneuvac3,x.age65yr,x.casthm1,x.drdxar1,x.rfsmok3} => {shingle2} | 0.16 | 0.53 | 1.79 | 2806 |
|  |  | {frenchf1,x.casthm1,x.drdxar1,x.rfsmok3} => {potatoe1} | 0.15 | 0.75 | 1.75 | 2612 |
|  |  | {pneuvac3,shingle2,x.casthm1,x.drdxar1,x.rfsmok3} => {x.age65yr} | 0.16 | 0.88 | 1.54 | 2806 |
|  |  | {diabete3,x.casthm1,x.drdxar1,x.rfhype5,x.rfsmok3} => {diffwalk} | 0.15 | 0.71 | 1.51 | 2702 |
|  |  | {diabete3,x.drdxar1,x.rfhype5,x.rfsmok3} => {diffwalk} | 0.16 | 0.71 | 1.51 | 2731 |
| Diabetes | 'diabete3' | {diffwalk,x.casthm1,x.rfchol1,x.rfhype5,x.rfsmok3} => {diabete3} | 0.14 | 0.58 | 1.53 | 2533 |
|  |  | {diabete3,x.casthm1,x.drdxar1,x.rfhype5,x.rfsmok3} => {diffwalk} | 0.15 | 0.71 | 1.51 | 2702 |
|  |  | {diabete3,x.drdxar1,x.rfhype5,x.rfsmok3} => {diffwalk} | 0.16 | 0.71 | 1.51 | 2731 |
|  |  | {diabete3,x.casthm1,x.drdxar1,x.rfbmi5,x.rfsmok3} => {diffwalk} | 0.15 | 0.71 | 1.51 | 2545 |
|  |  | {diffwalk,x.rfbmi5,x.rfhype5,x.rfsmok3} => {diabete3} | 0.17 | 0.57 | 1.51 | 2896 |
|  |  | {diffwalk,x.casthm1,x.rfbmi5,x.rfhype5,x.rfsmok3} => {diabete3} | 0.16 | 0.57 | 1.51 | 2866 |
|  |  | {diabete3,x.casthm1,x.drdxar1,x.rfsmok3} => {diffwalk} | 0.17 | 0.70 | 1.5 | 3032 |
|  |  | {diabete3,x.casthm1,x.drdxar1,x.rfbmi5} => {diffwalk} | 0.15 | 0.70 | 1.49 | 2572 |
|  |  | {pneuvac3,x.casthm1,x.rfbmi5,x.rfchol1,x.rfhype5,x.rfsmok3} => {diabete3} | 0.14 | 0.56 | 1.49 | 2532 |
|  |  | {diabete3,x.casthm1,x.drdxar1,x.rfhype5} => {diffwalk} | 0.16 | 0.70 | 1.48 | 2733 |
| Cancer | 'chcocncr' | {x.age65yr,x.casthm1} => {chcocncr} | 0.15 | 0.27 | 1.2 | 2697 |
|  |  | {chcocncr,x.casthm1,x.rfsmok3} => {x.age65yr} | 0.15 | 0.68 | 1.2 | 2610 |
|  |  | {chcocncr,x.casthm1,x.rfsmok3} => {pneuvac3} | 0.16 | 0.73 | 1.13 | 2775 |
|  |  | {pneuvac3,x.casthm1,x.rfsmok3} => {chcocncr} | 0.16 | 0.25 | 1.11 | 2775 |
|  |  | {x.casthm1,x.drdxar1} => {chcocncr} | 0.15 | 0.24 | 1.05 | 2551 |
|  |  | {chcocncr,fruit2,x.casthm1} => {x.rfsmok3} | 0.17 | 1.00 | 1.03 | 2918 |
|  |  | {chcocncr,pneuvac3,x.casthm1} => {x.rfsmok3} | 0.16 | 0.99 | 1.03 | 2775 |
|  |  | {x.casthm1,x.rfhype5,x.rfsmok3} => {chcocncr} | 0.16 | 0.23 | 1.02 | 2831 |
|  |  | {chcocncr,x.casthm1} => {x.rfhype5} | 0.17 | 0.74 | 1.02 | 2921 |
|  |  | {chcocncr,x.rfbmi5} => {x.rfsmok3} | 0.15 | 0.98 | 1.01 | 2699 |

^a^ We using the variable code for statistic of each variable, the meaning of code shown in supplement 1.
