# Supplementary material for: Detecting Lifestyle Risk Factors for Chronic Kidney Disease With Comorbidities: Association Rule Mining Analysis of Web-Based Survey Data
Source: J Med Internet Res. 2019 Dec 10;21(12):e14204. doi: 10.2196/14204 (PMC6930505; doi:10.2196/14204)
Supplement: Multimedia Appendix 4 [file jmir_v21i12e14204_app4.docx]

**Lifestyle Related Rules from SemmedDB**

| **Lifestyle Word List** | **Top 20 Rules** | **Lift** | **Count** |
| --- | --- | --- | --- |
| "Abuse of laxatives", "Adjustment Disorders", "Alcohol consumption", "Anorexia Nervosa", "Arsenic", "Beverages", "Cacodylate", "Cadmium", "Carbonated drink", "Carcinogens", "Cigarette smoke", "Cigarette Smoking", "Cobrotoxin", "Cocaine", "Cognition Disorders", "COGNITIVE DEFICIT", "Confusion", "Cooking", "Dairy Products", "defensiveness", "Delirium", "Dementia", "Dependence", "Depressive disorder", "Depressive Syndrome", "Diet", "Dietary Fiber", "Diethylstilbestrol", "Dimethylnitrosamine", "Direct Lytic Factors", "Driving While Intoxicated", "Drug abuse", "Dry food", "Dysfunctional coping using self defense mechanisms", "Emotional distress", "Environmental Tobacco Smoke", "Exercise", "Female sexual dysfunction", "ferric nitrilotriacetate", "Fitness", "Food", "forgetting", "Fruit juice", "Fruit", "Health behavior", "heavy drinking", "Herbicides", "Hydrogen Sulfide", "Illicit Drugs", "intravenous drug use", "Leisure physical activity", "Major Depressive Disorder", "Maladjustment", "melamine", "Memory impairment", "Mental Depression", "Mental disorders", "Methylguanidine", "Moderate Exercise", "moonshine", "Mutagens", "naphtha", "Nuts", "Obsessive-Compulsive Disorder", "Opiates", "Opioids", "Pain Disorder", "participation", "Performance", "Pesticides", "Physical activity", "Poultry Meat", "Processed meat", "Psychiatric problem", "Psychogenic polydipsia", "Psychosexual Disorders", "PSYCHOSOCIAL IMPAIRMENT", "Pulse vegetables", "Reading", "Regular exercise", "regular physical activity", "Relaxation", "renal toxin", "Rest", "Rotenone", "Schizophrenia", "Selenium", "Smoking", "Somatic delusion disorder", "Speaking", "Sports", "Tetrachlorodibenzodioxin", "Therapeutic diets", "toxicant", "Toxin", "travel", "Virulence Factors", "Walking", "Warfarin", "Wheat flour", "Pregnant Women", "Deafness", "Ability to concentrate", "Unable to concentrate", "Reduced concentration", "Sedentary lifestyle", "Physical function", "Former smoker", "Smoker", "Sedentary", "salt intake", "Sleeplessness", "Sleep Apnea Syndromes", "Dietary sodium intake", "SODIUM INTAKE", "hypercholesterolemia", "Overweight", "Muscle strength", "Dietary protein intake", "Dietary Fats", "Dietary salt intake", "Malnutrition", "Anorexia", "Carbohydrates", "Glucose Intolerance", "Iron deficiency", "Zinc deficiency", "Folic Acid Deficiency", "Vitamin B 6 Deficiency", "Vitamin D Deficiency", "Vitamin B 12 Deficiency", "Vitamin K Deficiency", "Calcium intake", "Selenium deficiency", "Dehydration", "PROTEIN INTAKE", "Phosphorus intake", "POTASSIUM INTAKE", "Stress", "Chronic Stress", "Depressed mood" | {Heat Stress Disorders} => {Dehydration} | 699.67 | 2 |
